# Editorial: Highlights in HIV and STIs, 2021/2022

**DOI:** 10.3389/frph.2022.1116800

**Published:** 2023-01-12

**Authors:** Matthew S. Hogben, Garumma T. Feyissa

**Affiliations:** ^1^ Division of STD Prevention, Centers for Disease Control and Prevention, Atlanta, GA, United States; ^2^ Dornsife School of Public Health, Drexel University, Philadelphia, PA, United States

**Keywords:** HIV, STI (science, technology and innovation), treatment, implementability, program evaluation

**Editorial on the Research Topic** Highlights in HIV and STIs, 2021/2022

The collection of HIV and STI prevention and care articles in this Research Topic is drawn from three countries on two continents. Each appears on its own merit, and, as a group, they also illustrate important current attributes of current HIV/STI prevention. In this summary, we draw out these attributes as well as speak briefly about the overarching research principles these articles embody.

Two of the articles in this collection are centered around specimen self-collection, effectively expanding service provision and use in sexual health. Pierz et al. ascertain the acceptability of self-sampling for cervical cancer screening among women in Limbe, Cameroon (some of whom were living with HIV). Women in this study did collect their own specimens (vaginal/cervical) in a private room onsite at a hospital. An extensive array of focus group discussions and in-depth interviews revealed concerns about pain and the ability to collect specimens. Participants in the study also spoke about the inhibiting presence of stigma related to HIV status, whether or not they were living with HIV themselves. Hence, it is critical to address stigma and discrimination in healthcare settings. The second article (Leenen et al.) also gathered formative data about self-sampling, this time among men who have sex with men (MSM) in the Netherlands. The authors present an intervention mapping approach to increase self-sampling, this time outside of clinical settings, beginning with assessment data from MSM to guide the mapping process and increase patient engagement. Leenen et al. frame their article around increasing opportunities to test for HIV in a resource-rich country. Despite different focus populations, different pathogens, and the substantive national differences between Cameroon and the Netherlands, Leneen et al. and Pierz et al. find similar issues pertaining to stigma and privacy as barriers to testing. This underscores the necessity of designing strategies to reduce stigma and discrimination for effective prevention and control of HIV/AIDS and STIs, as well as promotion and acceptance of sexual health services. For example, Feyissa et al. suggest an evidence-informed guideline to reduce stigma and discrimination in healthcare settings so that stigma reduction efforts will be sustained by making them part of the routine tasks of healthcare institutions and the professional responsibilities of healthcare workers (1). Both articles used a socio-ecological framework, explicitly in Pierz et al. and implicitly in Figure 1 in Leenen et al., to guide work to improve implementation through a theoretical frame.

Of the remaining two articles, Slurink et al. used surveillance data to detect patterns in recent HIV infection, expanding the utility of specimens if not the manner of sampling. In a demonstration of the importance of sexual health clinics, the authors find a higher proportion of recent infections (27%) in these clinics, compared to other testing sites (5%–15%). For those testing in this Dutch study at least, clinics might serve as a particular resource for people at the highest risk of acquiring HIV. Given the salience of recent infection to transmission (2), sexual health clinics would then be central to identifying and preventing transmission in populations. The final article in this collection, Anyanwu et al., examines the relationship between herbal remedies and substance use (specifically, alcohol use and smoking tobacco) and liver function in HIV-infected patients in care in Port Harcourt, Nigeria. Both alcohol use and smoking were associated with higher liver enzyme levels, although herbal remedies used in traditional medicine were not. This analysis reminds us that care for HIV patients (the patients in this study had received antiretroviral therapy for at least one year) requires a complex and multifaceted approach.

Along with a theme of expanded testing coverage or expanded use of test results for public health benefit, the studies in these articles often facilitated the engagement of the focus populations in their own care or support as an initial tactic. The use of qualitative assessments in both Pierz et al. and Leenen et al. are examples, as is the explicit use of assessment data as an initial guide to intervention mapping in the second study. Qualitative and quantitative assessments garner information about populations, disease epidemiology, and social environments, providing the context essential to formulating successful interventions (3).

While discussing engaging focus populations, we can also draw upon the examples of Anyanwu et al. and Pierz et al. to note that people either living with HIV or vulnerable to contracting HIV have healthcare needs that extend beyond HIV. In fact, given that many such individuals are members of socially and economically marginalized populations, their needs are not even confined to healthcare. Many sexual health and STI/HIV prevention programs also offer linkage to social services. Finally, this linkage is not just for people; Slurink et al. demonstrate with their use of specimens to ascertain recent HIV infection that patient health care needs (testing) can be linked to data to inform public health needs (prevention of transmission at the population level).

We conclude here with a few thoughts based on these articles about frameworks for research. This collection contains observational studies, but at least one is clearly designed for implementation work (*via* intervention mapping), and the remainder have clear paths from ascertainment and discovery to intervention, translation, and implementation. Leenen et al. even measure the effects of *prior* translation efforts – guideline restrictions on testing opportunities – on barriers to prospective impact. A path from observation through intervention testing to implementation and impact is always an advantage in almost any stage of research; here, we allude to just such a model specifically incorporating prevention programs such as those in the articles we have summarized. Aral, Blanchard, and colleagues formulated an approach they named *program science*, in which strategic planning is combined with scientifically supported program implementation and then monitoring (4). This integrated approach incorporates implementation and operational research aimed at testing in “real world” settings, and the cyclical nature of the program drives further improvements aimed at population health impact. Any of the studies in this Research Topic would fit well as part of a program science agenda.
